# Cardiomyocyte cohesion is increased after ADAM17 inhibition

**DOI:** 10.3389/fcell.2023.1021595

**Published:** 2023-01-17

**Authors:** Maria Shoykhet, Jens Waschke, Sunil Yeruva

**Affiliations:** Chair of Vegetative Anatomy, Institute of Anatomy, Faculty of Medicine, Ludwig-Maximilian-University (LMU), Munich, Germany

**Keywords:** arrhythmogenic cardiomyopathy, desmoglein 2, ADAM17, p38MAPK, EGFR

## Abstract

A Disintegrin And Metalloprotease (ADAM) family proteins are involved in several cardiac diseases, and some ADAMs have been associated with cardiomyopathies. ADAM17 is known to cleave desmoglein 2 (DSG2), one of the proteins involved in the pathogenesis of arrhythmogenic cardiomyopathy (AC). Desmosomal stability is impaired in AC, an inheritable genetic disease, the underlying causes of which can be mutations in genes coding for proteins of the desmosome, such as DSG2, desmoplakin (DP), plakoglobin (PG), plakophilin 2 or desmocollin 2. Stabilizing desmosomal contacts can therefore be a treatment option. In the heart of the murine *Jup* ^−/−^ AC model, (*Jup* being the gene coding for PG) mice, elevated levels of p38MAPK, an activator of ADAM17, were found. However, ADAM17 levels were unaltered in *Jup* ^−/−^ mice hearts. Nonetheless, inhibition of ADAM17 led to enhanced cardiomyocyte cohesion in both *Jup* ^+/+^ and *Jup* ^−/−^ mice, and in HL-1 cardiomyocytes. Further, enhanced cohesion in HL-1 cardiomyocytes after acute inhibition of ADAM17 was paralleled by enhanced localization of DSG2 and DP at the membrane, whereas no changes in desmosomal assembly or the desmosomal complex were observed. In conclusion, acute inhibition of ADAM17 might lead to reduced cleavage of DSG2, thereby stabilizing the desmosomal adhesion, evidenced by increased DSG2 and DP localization at cell borders and eventually cardiomyocyte cohesion. We believe that similar mechanisms exist in AC.

## 1 Introduction

Members of the A Disintegrin And Metalloprotease (ADAM) family are transmembrane proteins with proteolytic activity, that can be involved in cancers, cardiac diseases, diseases of the central nervous system or the liver (Zhang et al., 2016). So far, four ADAMs (ADAMs 10, 12, 15, and 17) have been associated with cardiomyopathies (Fedak et al., 2006). Out of these, ADAM17 is known to cleave desmoglein 2 (DSG2), a calcium-dependent adhesion protein (cadherin), which was found to be a key regulator of cardiomyocyte cohesion (Schinner et al., 2020; Shoykhet et al., 2020; Yeruva et al., 2020).

Today over 80 substrates of ADAM17, also known as tumor necrosis factor α (TNFα) converting enzyme (TACE) are known, including the epidermal growth factor (EGF), the transforming growth factor α (TGFα), the β_1_-adrenergic receptor, but also proteins involved in cell cohesion, i.e., DSG2 or collagen XVII (Santiago-Josefat et al., 2007; Wang et al., 2015; Zunke and Rose-John, 2017; Zhu and Steinberg, 2021). In H9C2 cardiomyocytes, ADAM17 was a key player driving hypertrophy (Shi et al., 2021). Increased ADAM17 activity was observed after myocardial infarction (MI) (Akatsu et al., 2003; Ghaderian et al., 2011; Guan et al., 2021), whereas ADAM17 inhibition decreased the amount of fibrosis after MI in mice (Guan et al., 2021). ADAM17 was increased in chronically ischemic mouse hearts as well as in ischemic rat hearts (Lu et al., 2012; Palaniyappan et al., 2013). Furthermore, ADAM17 expression was increased in peripheral blood mononuclear cells from heart failure patients, which correlated with TNFα-levels and was associated with severity of the condition (Satoh et al., 2004). Apart from that, increased ADAM17 activity was also found in patients with acute myocarditis, and hypertrophic cardiomyopathy or dilated cardiomyopathy (DCM), as well as in mice with diabetic cardiomyopathy (Satoh et al., 1999; Satoh et al., 2000; Fedak et al., 2006; Xue et al., 2022). A deficiency of tissue inhibitor of metalloproteases 3 (TIMP3), a physiological inhibitor of ADAM17, can result in DCM (Fedak et al., 2004). Finally, in a *Dsg2* ^−/−^ murine model for arrhythmogenic cardiomyopathy (AC), ADAM17 levels were increased in the hearts of 2 week old mice (Ng et al., 2021).

AC is a genetic disorder that leads to a fibro-fatty replacement of cardiac muscle tissue. Untreated, AC can lead to sudden cardiac death. Underlying causes of the disease include mutations in genes coding for proteins of the desmosome, i.e., DSG2, desmocollin 2 (DSC2), plakophilin 2 (PKP2), desmoplakin (DP) or plakoglobin (PG), but also proteins not associated with the desmosome can be affected. Apart from weakened cardiomyocyte cohesion, cardiomyocyte apoptosis and necrosis, fibro-fatty replacement of the myocardium, and arrhythmias, AC also can lead to inflammation of the cardiac tissue, which damages the affected tissue further (Basso et al., 1996; Campuzano et al., 2012; van der Voorn et al., 2020). The inflammatory response differs from patient to patient, but can include increased levels of interferon γ, interleukin 1β, the nuclear factor κB (NF-κB), as well as TNFα (Chelko et al., 2019; Lin et al., 2021).

Inhibition of ADAM17 might be beneficial in the setting of AC as seen in other cardiovascular diseases (Saftig and Reiss, 2011). Increased TNFα levels were observed in some AC patients and were correlated with disease progression. Inhibition of ADAM17 might decrease soluble TNFα levels. Since ADAM17 can shed proteins involved in cellular cohesion, and cellular contacts are often disturbed in AC, inhibiting this protease might protect cell-cell contacts in AC. Therefore, we investigated the effect of inhibition of ADAM17 on cellular cohesion using a murine AC model and HL-1 cardiomyocytes.

## 2 Materials and methods

### 2.1 Cell culture

Immortalized murine cardiomyocytes (HL-1 cardiomyocytes), provided by William Claycomb (LSU Health Sciences Center, New Orleans, United States), were cultivated as described before (Shoykhet et al., 2020). For experiments, cells were cultured at 125,000 cells/cm^2^ for 4 days in medium without norepinephrine.

### 2.2 Murine AC model and cardiac slice cultures

Murine cardiac slice cultures from previously characterized 12 week old C57BL6/J wildtype as well as cardiomyocyte-specific *Jup* ^−/−^ mice were obtained as described before (Schinner et al., 2017; Shoykhet et al., 2020). Animal handling was in accordance with the guidelines from the Directive 2010/63/EU of the European Parliament and approved by the regional government of Upper Bavaria (Gz. ROB-55.2-2532. Vet_02-19-172).

### 2.3 Mediators and reagents

Samples were treated for 90 min with the ADAM17-inhibitor TAPI-1 (TAPI, Cayman Chemical, #18505, final concentration: 10 µM) dissolved in DMSO. For Ca^2+^-switch experiments, cells were depleted of Ca^2+^ using the Ca^2+^-chelator EGTA (VWR, #0732, final concentration: 5 mM) for 90 min. After changing the medium back to Ca^2+^-containing medium and incubation with the respective mediators, experiments were performed.

### 2.4 Lysate preparation

For Western blots, samples were washed with PBS on ice and lysed into SDS lysis buffer with protease (cOmplete^TM^ Protease Inhibitor Cocktail, Roche, #CO-RO) and phosphotase (PhosStop^TM^, Roche, #PHOSS-RO) inhibitors, scraped into reaction tubes and sonicated.

For Triton assays, cells were lysed into Triton buffer with protease and phosphatase inhibitors, by adding the buffer to the cells on a rocking platform for 20 min while on ice. The lysate was scraped into reaction tubes; separation of detergent soluble and insoluble fractions was achieved by centrifugation at 4°C for 10 min at 15,000 g in an Eppendorf 5430R centrifuge. After transferring the Triton soluble fractions to new tubes, the insoluble fraction was washed with Triton buffer, resuspended in SDS lysis buffer and sonicated.

For immunoprecipitations, cells were cultured in T75 flasks and after treatments lysed for 30 min on ice on a rocking platform into RIPA lysis buffer (10 mM Na_2_HPO_4_, 150 mM NaCl, 1% Triton X-100, 0.25% SDS, 1% sodium deoxycholate, pH = 7.2) containing protease and phosphatase inhibitors. The lysate was then transferred to gentleMACS™ M-tubes (Miltenyi Biotec, #130-093-236), and dissociated using the protein_01_01 program of the gentleMACS™ OctoDissociator (Miltenyi Biotec, #130-095-937).

When preparing lysates from murine cardiac slice cultures, slices were snap-frozen in liquid nitrogen after treatment and washing with TBS. Slices were transferred into SDS lysis buffer in gentleMACS™ M-tubes. For dissociation, the protein_01_01 program of the gentleMACS™ OctoDissociator was used.

The Pierce™ Protein Assay Kit (Thermo Fisher, #23225) was used to measure protein concentration according to the manufacturer’s protocol.

### 2.5 Western blot analyses

After lysate denaturation in Laemmli buffer for 10 min at 95°C, lysates were loaded on SDS-PAGE gels together with the PageRuler™ Plus Prestained Protein Ladder (Thermo Fisher, #26620). Transfer to nitrocellulose membranes (Thermo Fisher, #LC2006) was achieved using the wet-blot method. After blocking, membranes were incubated with primary antibodies overnight at 4°C: anti-ADAM17 (abcam, #ab39162), anti-DES (abcam, #32362), anti-DP (Progen, #61003), anti-DSG1/2 (Progen, #61002), anti-pEGFR845 (Cell Signaling, #2231), anti-pEGFR1068 (Cell Signaling, #2234), anti-EGFR (Santa Cruz, #373746), anti-pERK (Santa Cruz, #7383), anti-ERK (Cell Signaling, #9102), anti-N-CAD (BD Transduction, #610921), anti-p38MAPK (Cell Signaling, #9212), anti-pp38MAPK (Cell Signaling, #4511), anti-PG ((PG5.1) Progen #61005), anti-PKP2 (Progen, #651167), anti-phospho-Thr (Cell Signaling, #9386) and anti-α-tubulin ((DM1A), abcam, #7291) were used as primary antibodies. HRP-coupled secondary antibodies (Dianova, #111-035-045 and #115-035-068) and SuperSignal™ West Pico PLUS Chemiluminescent Substrate (Thermo Fisher, #34577) were used to develop membranes with an iBright™ FL1500 developer (Thermo Fisher). NoStain™ Protein Labelling Reagent (Thermo Fisher, #A44449) was used as loading control. Quantification was performed using the iBright™ Analysis Software (Invitrogen).

### 2.6 Dispase-based dissociation assays

For dispase-based dissociation assays, HL-1 monolayers were detached from well bottoms after washing with HBSS by adding Liberase-DH (Sigma-Aldrich, #5401054001), followed by dispase II (Sigma-Aldrich, #D4693) and incubation at 37°C, 5% CO_2_. After replacing the enzyme mix by HBSS once the monolayer was detached, MTT was added for better visibility and monolayers were subjected to mechanical stress by shaking at 1.31 g for 5 min on an orbital shaker (Stuart SSM5 orbital shaker). The higher the amount of the monolayer fragmentation after mechanical stress, the lower the cellular cohesion and *vice versa*. Murine cardiac slice dissociation assays were performed similarly; however, Liberase-DH and dispase II were added simultaneously for 30 min. After adding MTT, equal mechanical stress using an electrical pipette was applied, and the resulting mix with heart tissue fragments, single dissociated cardiomyocytes and cell debris was filtered using a 70 µm nylon membrane (PluriSelect, #43-10070-60). Pictures of the filtered cell suspension well were taken in gridded 96 well plates and stitched together using the AutoStich software (Brown and Lowe, 2007). The number of dissociated rod-shaped, MTT-stained viable cardiomyocytes was counted using ImageJ software and normalized to the respective controls.

### 2.7 Immunostainings

Cells were seeded on coverslips, fixed with 2% paraformaldehyde and permeabilized with 0.1% Triton X-100. After blocking with bovine serum albumin and normal goat serum, primary antibodies were added overnight at 4°C in a wet chamber. anti-DP (Progen, #61003), anti-DSG2 (Progen, #610121) and anti-N-CAD (BD Transduction Laboratories™, #610921) were used at 1:100 dilution. Fluorophore-coupled, species-matched secondary antibodies were added together with Phalloidin and DAPI (Phalloidin, 130 nM, Thermo Fisher #A12379 and DAPI, 0.5 μg/ml, #10236276001, Roche). For image acquisition, a Leica SP5 II confocal microscope (Leica, Mannheim, Germany) with a 63X oil objective and the LAS-AF software was used. Z-scans were performed at 0.25 µm thickness spanning the whole cell volume. Image analysis was performed using the ImageJ software. For colocalization analysis, a region of interest at the membranes was chosen and the amount of stained pixels in this area in the other channels was calculated (Supplementary Figure S3B).

### 2.8 Immunoprecipitation

1.5 mg of protein lysate was incubated with 1 µg of anti-DSG2/ADAM17 or control mouse IgG (Merck, #12-371) or rabbit IgG (Covalab, #pab01004-P) overnight at 4°C on a spinning wheel. Protein G Dynabeads™ (Thermo Fisher, #10004D) were used to pull down the complexes by adding the lysate-antibody mix to prewashed beads for 1 h at 4°C. Afterwards, beads were isolated and washed using a magnetic rack, by washing twice with wash buffer 1 (50 mM Tris-HCl, 150 mM NaCl, 0.1 mM EDTA, 0.5% Tween20, pH = 7.5), thrice with wash buffer 2 (100 mM Tris-HCl, 200 mM NaCl, 2 M Urea, 0.5% Tween20, pH = 7.5), and twice with 1% Triton X-100 in PBS. After resuspension in Laemmli buffer, the samples were denatured for 10 min at 95°C and the lysate was separated from the beads using a magnet and loaded on a SDS-PAGE gel for Western blot analyses.

### 2.9 Sulfo-EGS crosslinking

Sulfo-ethylene glycol bis(sulfosuccinimidyl succinate) (Sulfo-EGS, Thermo Fisher, #21566), a membrane-impermeable cross-linker, was used to analyze oligomerization of DSG2 at the cell membrane. To that end, after 90 min of TAPI-1 treatment, HL-1 cardiomyocytes were washed with ice-cold PBS. Then, 2 mM Sulfo-EGS in PBS was added for 30 min at room temperature. The reaction was stopped with TBS and cells were scraped into SDS lysis buffer and analyzed *via* Western blot.

### 2.10 Imaging and statistics

Adobe Photoshop CS5 and ImageJ softwares were used for image processing. For Western blot quantification, the iBright™ Analysis Software was used. GraphPad Prism 8 was used for statistical analysis of the data, using unpaired Student’s *t*-tests after outlier removal. Graphs are represented as mean ± standard deviation (SD). Data was normalized to the average control value in Western blots and dissociation assays in HL-1 cardiomyocytes. When analyzing dissociation assays performed in murine cardiac slices, normalization was performed to the respective control slice of the same mouse. Significance was assumed for *p* ≤ 0.05.

## 3 Results

### 3.1 ADAM17 inhibition by TAPI-1 induces positive adhesiotropy in a murine model for AC

**FIGURE 1:**
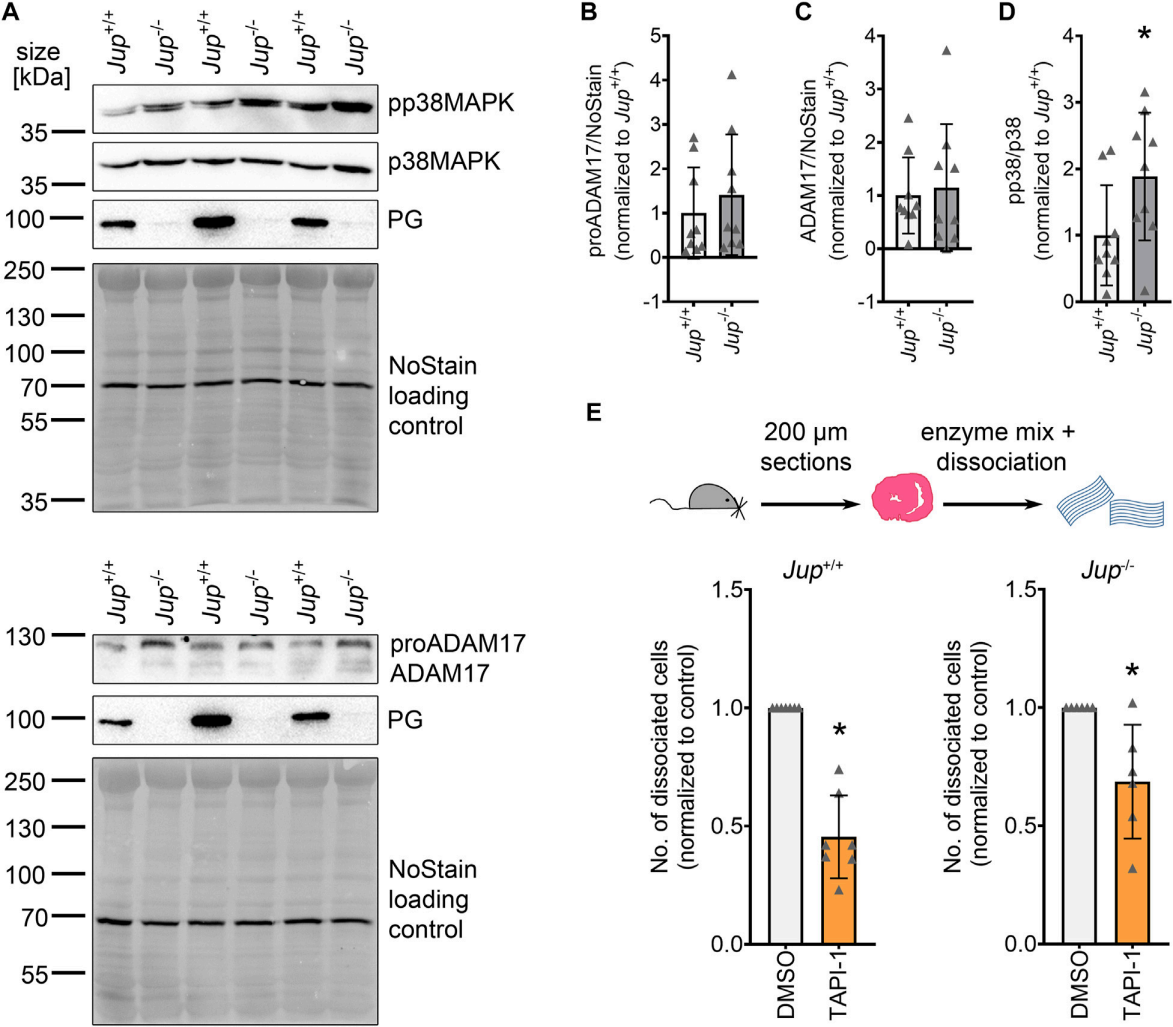
ADAM17 inhibition leads to positive adhesiotropy in *Jup* ^+/+^ and *Jup* ^−/−^ mice. **(A)** Representative Western blot showing protein expression of pp38MAPK, p38MAPK, ADAM17 and PG in *Jup* ^+/+^ and *Jup* ^−/−^ mice. NoStain Dye™ was used as loading control. **(B–D)**: Quantification of protein expression assessed by Western blots shown in **(A)**. **(B)** proADAM17 protein expression. **(C)** ADAM17 protein expression. **(D)** phosphorylation of p38MAPK. *p ≤ 0.05, unpaired Student’s *t*-test, *N* = 9 mice. **(E)** Dispase-based dissociation assay in murine cardiac slice cultures obtained from *Jup* ^+/+^ and *Jup* ^−/−^ mice upon inhibition of ADAM17 by TAPI-1. *p ≤ 0.05, unpaired Student’s *t*-test, *N* = 7 for *Jup* ^+/+^ and *N* = 6 for *Jup* ^−/−^ mice.

To investigate whether ADAM17 plays a role in AC, we assessed protein levels of ADAM17 and p38MAPK activation, a known activator of ADAM17, in *Jup* ^+/+^ mice as well as in the *Jup* ^−/−^ murine AC model. ADAM17 protein levels were variable, though not significantly increased in *Jup* ^−/−^ mice. Phosphorylation of p38MAPK was increased in *Jup* ^−/−^ mice as compared to *Jup* ^+/+^ controls (Figures 1A–D). However, we did not observe increased ADAM17 phosphorylation in *Jup* ^−/−^ mice (Supplementary Figure S1). Next, we performed dispase-based dissociation assays murine cardiac slices obtained from *Jup* ^+/+^ or *Jup* ^−/−^ mice. For the dissociation assay, murine cardiac slices were treated with dispase II and liberase-DH and then subjected to mechanical stress with an electrical pipette, resulting in dissociated cardiomyocytes. The amount of dissociated cardiomyocytes served as an inverse measure for cardiomyocyte cohesion. In *Jup* ^+/+^, as well as in *Jup* ^−/−^ mice, ADAM17 inhibition by TAPI-1 led to an increase in cardiomyocyte cohesion, which we term as positive adhesiotropy (Figure 1E).

These data suggested that inhibition of ADAM17 might be beneficial for cardiomyocyte cohesion in AC.

### 3.2 ADAM17 inhibition leads to increased cellular cohesion in HL-1 cardiomyocytes without changes in EGF receptor (EGFR) signaling

**FIGURE 2:**
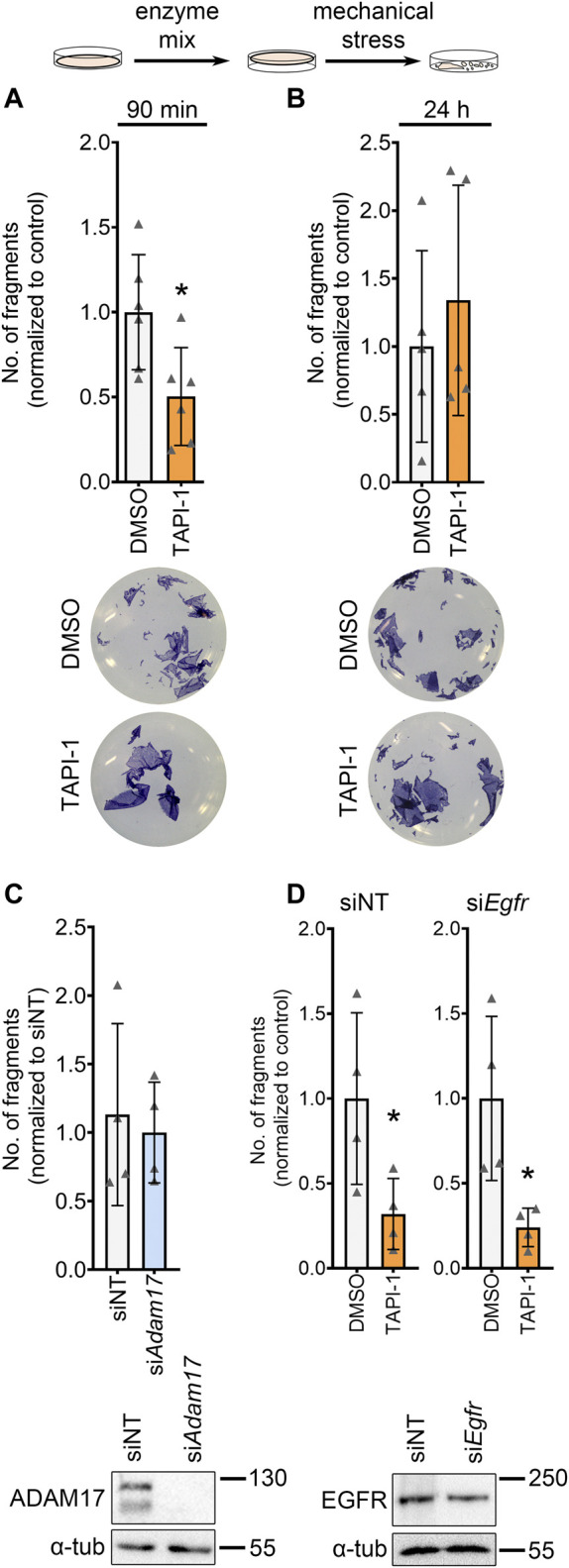
ADAM17 inhibition leads to positive adhesiotropy in HL-1 cardiomyocytes independent of EGFR. **(A, B)** Dispase-based dissociation assays in HL-1 cardiomyocytes upon inhibition of ADAM17 by TAPI-1, with representative pictures of the wells, **(A)** inhibition of ADAM17 for 90 min, *N* = 6 biological replicates. **(B)** inhibition of ADAM17 for 24 h, *N* = 5 biological replicates. *p ≤ 0.05, unpaired Student’s *t*-test. **(C)** Dispase-based dissociation assay in HL-1 cardiomyocytes after knockdown of *Adam17* by siRNA. Knockdown efficiency was confirmed by Western blot. *p ≤ 0.05, unpaired Student’s t-test, *N* = 4 biological replicates. **(D)** Dispase-based dissociation assay in HL-1 cardiomyocytes after knockdown of *Egfr* by siRNA and treatment with TAPI-1 for 90 min. Knockdown efficiency was confirmed by Western blot, representative images are shown below the respective bar graphs. *p ≤ 0.05, unpaired Student’s *t*-test, *N* = 4 biological replicates.

To further investigate the role of ADAM17 inhibition on cardiomyocytes, we switched to the HL-1 cardiomyocyte cell culture model. Firstly, we performed dispase-based dissociation assays in HL-1 cardiomyocytes. To that end, after respective treatments, HL-1 cardiomyocyte monolayers were detached from the well bottom using dispase II and liberase-DH and subjected to mechanical stress, leading to a fragmentation of the cell monolayer. The number of fragments were counted and taken as an indirect inverse measure of cardiomyocyte cohesion. Upon inhibition of ADAM17, we observed a decreased fragmentation of the cellular monolayers after mechanical stress, thus, an increase in cellular cohesion (Figure 2A). We confirmed that the observed effect of TAPI-1 is not an off-target effect by knocking down *Adam17* in HL-1 cardiomyocytes using siRNA and treating with TAPI-1 (Supplementary Figures 2A, B). Upon knockdown of *Adam17* using siRNA or application of the ADAM17 inhibitor for 24 h, we did not observe a change in cellular cohesion as compared to siNT (non-target) or vehicle treated HL-1 cardiomyocytes, respectively (Figures 2B, C). Since ADAM17 can transactivate the EGFR by EGF shedding, and EGFR inhibition can also lead to positive adhesiotropy (Shoykhet et al., 2022), we assessed whether the TAPI-1-induced positive adhesiotropy requires EGFR. However, we did not observe changes in EGFR signaling (Supplementary Figures 2C–F). Furthermore, TAPI-1 was still effective to induce positive adhesiotropy when EGFR protein levels were decreased by siRNA-mediated *Egfr* knockdown (Figure 2D and Supplementary Figure S2G).

These data indicate that positive adhesiotropy induced by ADAM17 inhibition might not be mediated through EGFR.

### 3.3 ADAM17 inhibition increases DSG2 localization at the membrane

**FIGURE 3:**
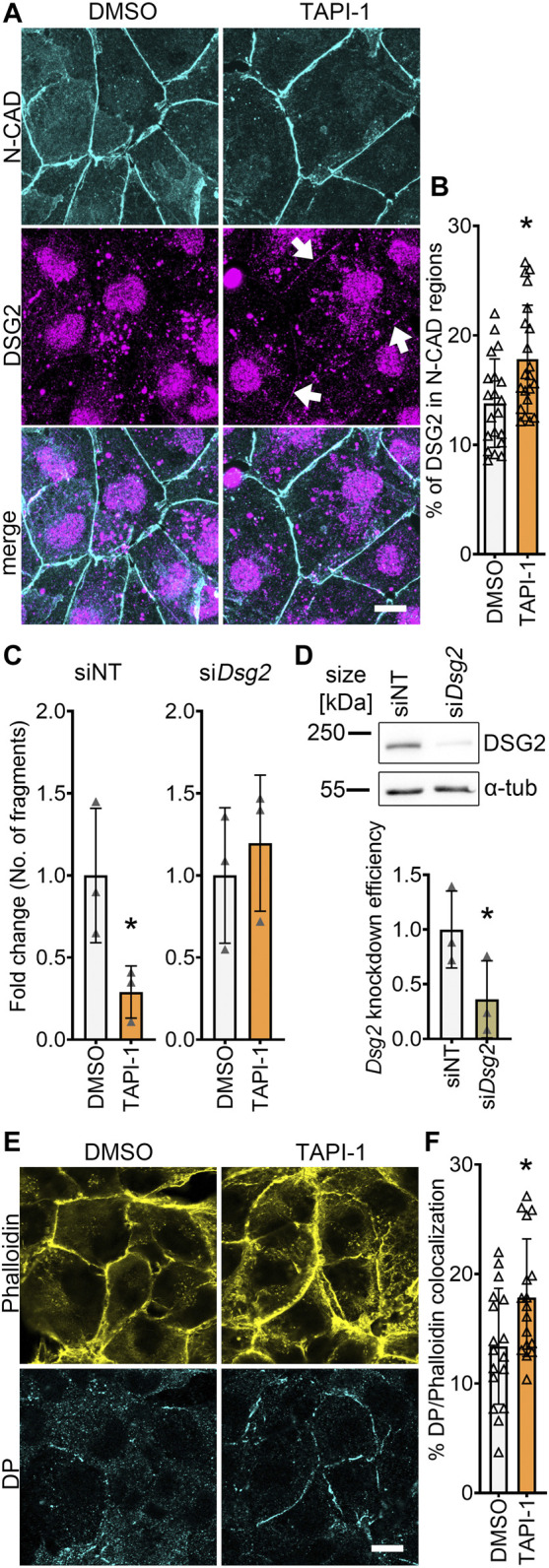
ADAM17 inhibition leads to enhanced localization of DSG2 and N-CAD at the cell borders. **(A)** Maximum projections of immunostainings in HL-1 cardiomyocytes for N-CAD and DSG2 showing an increase of DSG2 (white arrows) at the cell borders after 90 min of TAPI-1 treatment. Z-scans spanning the whole cell volume, z-steps = 0.25 µm. Scale bar: 10 µm. **(B)** Quantification of colocalization of N-CAD and DSG2 in HL-1 cardiomyocytes, *p ≤ 0.05, unpaired Student’s *t*-test. Each data point represents one image, *N* = 8 biological replicates. **(C)** Dispase-based dissociation assay in HL-1 cardiomyocytes after knockdown of *Dsg2* by siRNA and treatment with TAPI-1 for 90 min *p ≤ 0.05, unpaired Student’s *t*-test, *N* = 3 biological replicates. **(D)** Representative Western blot confirming *Dsg2* knockdown with quantification. *p ≤ 0.05, paired Student’s *t*-test, *N* = 3 biological replicates **(E)** Immunostaining of DP in HL-1 cardiomyocytes after 90 min of TAPI-1 treatment using Phalloidin as membrane marker. Scale bar: 10 µm. **(F)** Quantification of colocalization of DP and Phalloidin. *p ≤ 0.05, unpaired Student’s *t*-test. Each data point represents one image, *N* = 5 biological replicates.

To get insights on how ADAM17 inhibition led to positive adhesiotropy in HL-1 cardiomyocytes, we performed Triton X-100-assays to analyze the cytoskeletal and non-cytoskeletal fractions of the desmosomal proteins. We did not observe changes in desmosomal protein localization after ADAM17 inhibition between Triton X-100 insoluble (cytoskeletal bound) proteins and Triton X-100 soluble (not cytoskeletal bound) proteins (Supplementary Figure S3A). However, immunostaining performed for N-CAD and DSG2, revealed no changes in N-CAD staining, whereas DSG2 localization at the membrane was increased after ADAM17 inhibition (Figures 3A, B). Quantification of protein localization at the membrane is explained in the methods section and exemplified in Supplementary Figure S3B. To assess whether positive adhesiotropy upon inhibition of ADAM17 is mediated by DSG2, we knocked down *Dsg2* and inhibited ADAM17. TAPI-1 was not effective in enhancing cardiomyocyte cohesion upon *Dsg2* knockdown (Figures 3C, D).

Since we observed an increase in DSG2 localization at the membrane, we next assessed whether DP, which provides the cytoskeletal anchorage to desmosomes, was affected by inhibition of ADAM17. Indeed, we found an increase in DP localization at the cell borders after inhibition of ADAM17 (Figures 3E, F).

Together, these data suggests that acute ADAM17 inhibition enhances the localization of DSG2 and DP at the cell borders and that DSG2 is a key driver of positive adhesiotropy upon ADAM17 inhibition.

### 3.4 No alterations in the desmosomal complex upon inhibition of ADAM17

**FIGURE 4:**
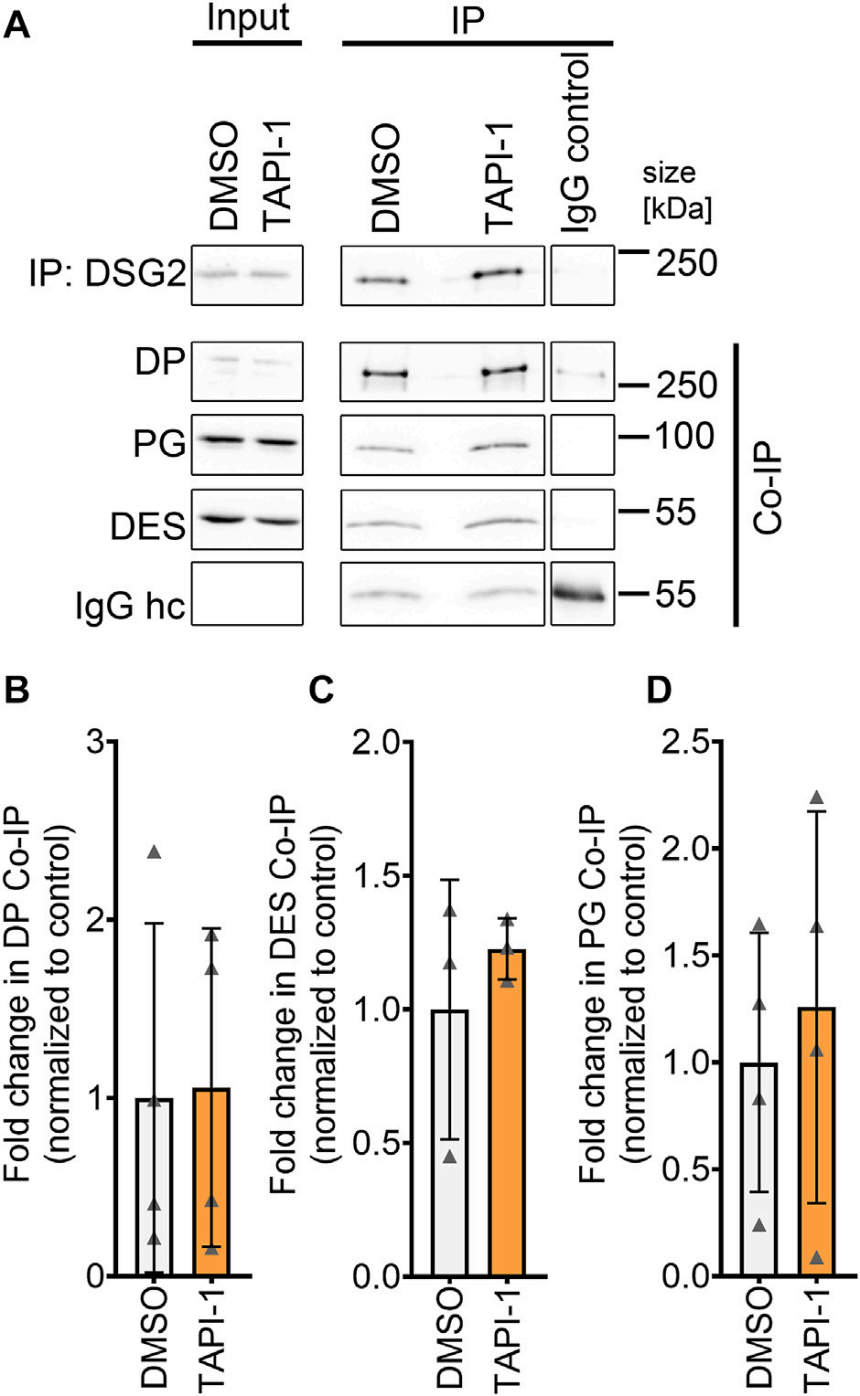
No change in desmosomal complex upon inhibition of ADAM17 by TAPI-1. **(A)** Representative Western blots for immunoprecipitation of DSG2 with quantification of co-immunoprecipitation (normalized to DSG2 pulldown) of **(B)** DP, **(C)** DES and **(D)** PG. IgG heavy chain (IgG hc) served as loading control for immunoprecipitated samples, *p ≤ 0.05, unpaired Student’s *t*-test, *N* = 4 biological replicates.

Next, we assessed whether increased DP and DSG2 localization at the membrane was paralleled by changes in the desmosomal complex. Therefore, we immunoprecipitated DSG2 and assessed the levels of other proteins of the desmosome which are in complex with DSG2. Immunoprecipitation of DSG2 revealed no changes in the desmosomal complex upon ADAM17 inhibition (Figures 4A–D).

### 3.5 The increase in cardiomyocyte cohesion is not caused by enhanced desmosomal assembly

**FIGURE 5:**
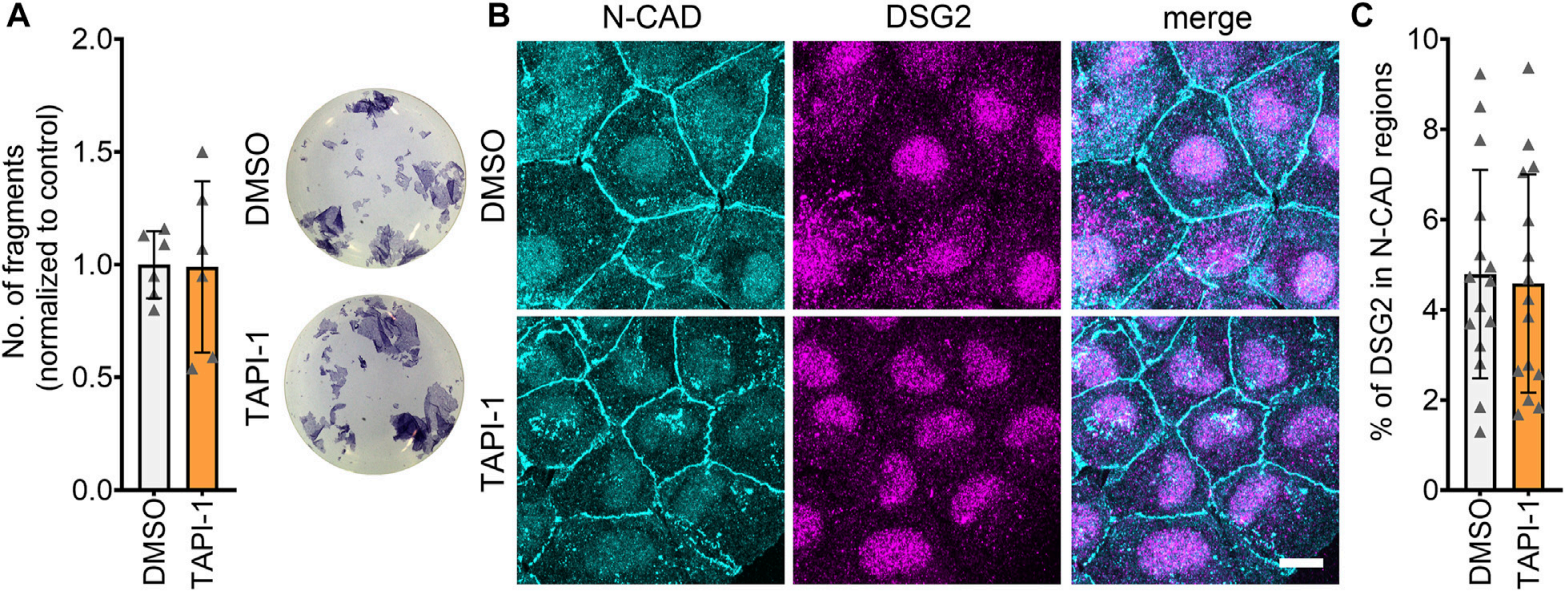
Desmosomal assembly is not enhanced upon inhibition of ADAM17. **(A)** Dispase-based dissociation assays in HL-1 cardiomyocytes after a Ca^2+^-switch followed by treatment with TAPI-1 for 90 min with representative pictures of the wells. *N* = 8, unpaired Student’s *t*-test, *p ≤ 0.05. **(B)** Maximum projections of immunostainings in HL-1 cardiomyocytes for N-CAD and DSG2 showing no changes in N-CAD and DSG2 localization after a Ca^2+^-switch followed by TAPI-1 treatment. Z-scans spanning the whole cell volume, z-steps = 0.25 µm. Scale bar: 10 µm. **(C)** Quantification of colocalization of N-CAD and DSG2 in HL-1 cardiomyocytes, *p ≤ 0.05, unpaired Student’s *t*-test. Each data point represents one image, *N* = 5 biological replicates.

One of the mechanisms that can lead to increased cellular cohesion is enhanced desmosomal assembly, which can be assessed by means of a Ca^2+^-switch assay. During a Ca^2+^-switch, cells are depleted of Ca^2+^ by treating with the Ca^2+^-chelator EGTA for 90 min. The Ca^2+^-depletion leads to a disruption of desmosomes, since desmosomal contacts are Ca^2+^-dependent. After Ca^2+^-repletion, desmosomes are reassembled, and upon addition of mediators, their effect on the desmosomal assembly can be assessed. Ca^2+^-switch assays did not reveal any alterations in cellular cohesion upon inhibition of ADAM17, indicating that acute ADAM17 inhibition does not lead to enhanced desmosomal assembly (Figure 5A). These findings were supported by no changes in the localization of N-CAD and DSG2 after a Ca^2+^-switch and ADAM17 inhibition (Figures 5B, C). Depletion of Ca^2+^ leads to desmosome disassembly resulting in a very low DSG2 localization at the membrane. Since ADAM17 inhibition-mediated positive adhesiotropy was not mediated by enhanced desmosomal assembly, we hypothesized that reduced cleavage of DSG2 might lead to reduced disassembly of desmosomes. However, we were neither able to detect cleaved fragments of DSG2 in the conditioned medium nor intracellular DSG2 fragments in the cell lysates (data not shown). We therefore performed EGS linking experiments. We observed a trend of increased DSG2 oligomer formation at the cell membrane after ADAM17 inhibition, however, this trend was not significant (Supplementary Figures S3C, D).

All in all, these data suggest that enhanced cellular cohesion after inhibition of ADAM17 was independent of desmosomal assembly.

## 4 Discussion

ADAM17, one of the first discovered sheddases, is known to cleave a plethora of proteins involved in inflammation, cell-to-cell communication, cellular signaling, and cell adhesion (Calligaris et al., 2021). ADAM17 was shown to cleave DSG2, one of the major proteins involved in cardiomyocyte cohesion (Schinner et al., 2017; Shoykhet et al., 2020; Yeruva et al., 2020). But the functional relevance of DSG2 cleavage in cardiomyocytes and its relevance to AC pathogenesis was not studied yet. Therefore, in this study we investigated the functional relevance of cleavage events by ADAM17 *via* inhibiting ADAM17 in *Jup* ^+/+^ mice and the *Jup* ^−/−^ murine AC model, as well as HL-1 cardiomyocytes. Here we show that acute inhibition of ADAM17 led to increased cardiomyocyte cohesion in *Jup* ^+/+^ and *Jup* ^−/−^ mice and in HL-1 cardiomyocytes. In HL-1 cardiomyocytes, ADAM17 inhibition-induced positive adhesiotropy was independent of EGFR and showed an increased localization of DSG2 and DP at the cell borders. Ca^2+^-switch experiments as well as immunoprecipitation experiments revealed that the increased cellular cohesion was not mediated by an enhanced desmosomal assembly or a strengthened desmosomal complex.

**FIGURE 6:**
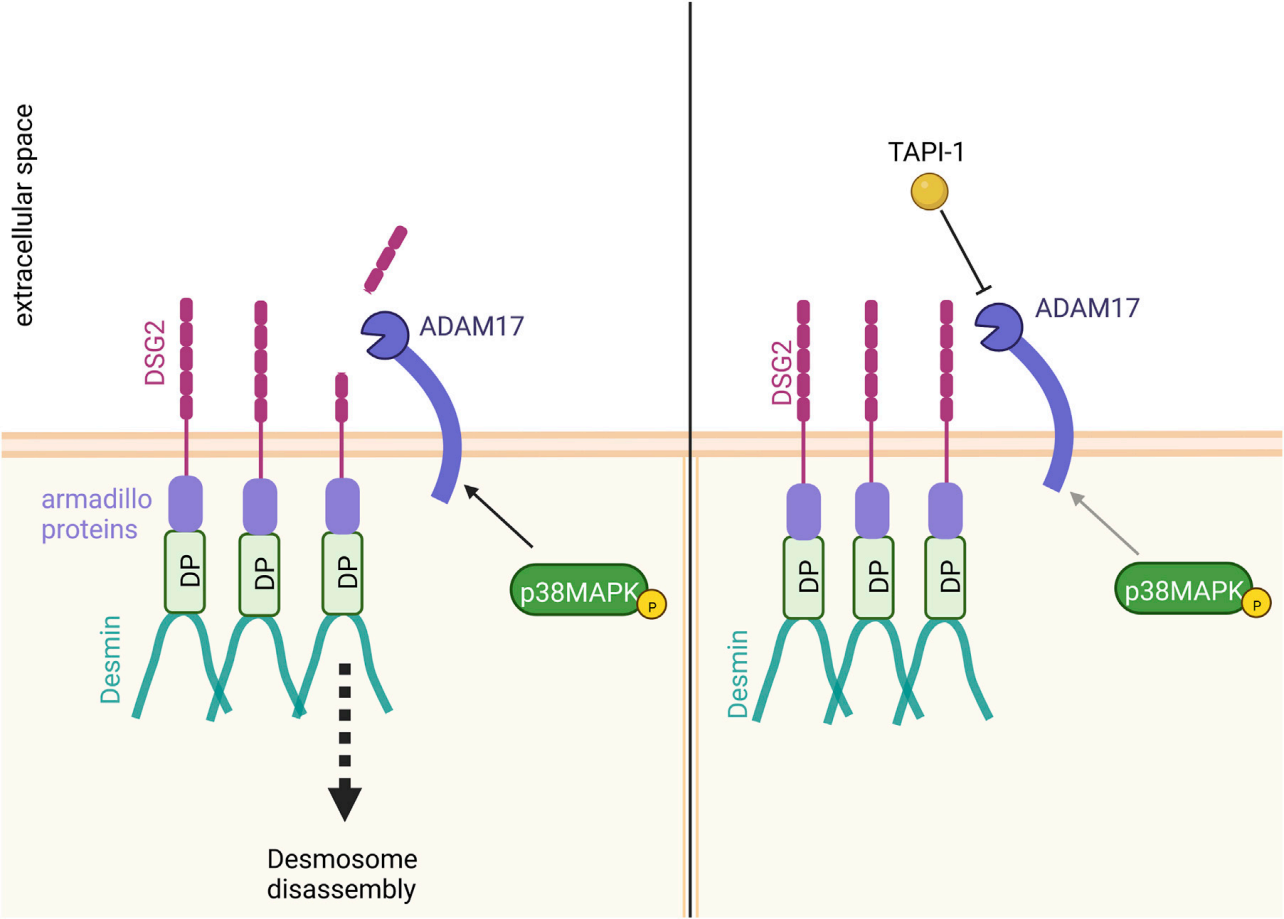
Proposed mechanism of action. Inhibition of ADAM17 by TAPI-1 enhanced the localization of DP and DSG2 at the membrane. Since we observed no changes in desmosomal assembly or the desmosomal complex upon inhibition of ADAM17, we hypothesize that the increase in cardiomyocyte cohesion upon inhibition of ADAM17 could result from reduced DSG2 cleavage. In contrast, without ADAM17 inhibition, basal ADAM17 activity leads to DSG2 cleavage with subsequent desmosome disassembly.

It has been shown that ADAM17 can cleave DSG2 (Bech-Serra et al., 2006; Klessner et al., 2009; Wang et al., 2015). We therefore hypothesized that the increase in cardiomyocyte cohesion upon inhibition of ADAM17 could result from a reduced DSG2 cleavage (Figure 6). Immunostainings supported this hypothesis, where we observed an increase of DSG2 and DP localization at the cell membrane after ADAM17 inhibition in HL-1 cardiomyocytes. However, we were not able to detect cleaved DSG2 fragments. It was previously shown that DSG2 cleavage by ADAM17 leads to DSG2 internalization, resulting in desmosome disassembly (Klessner et al., 2009). Therefore, upon ADAM17 inhibition, DSG2 cleavage was prevented leading to the retention of DSG2 at the membrane which further resulted in enhanced DP protein localization at the membrane. Furthermore we observed that neither known signaling pathways inducing positive adhesiotropy, nor desmosomal assembly or the desmosomal complex were changed. A possible mechanism by which enhanced desmosomal stability and stronger cardiomyocyte cohesion were achieved was by reduced DSG2 cleavage upon inhibition of ADAM17, leading to the accumulation of DSG2 at cell borders.

In keratinocytes, transactivation of the EGFR by ADAM17 increased cell migration (Maretzky et al., 2011), implying weakened cellular cohesion. Our data indicates that though ADAM17 inhibition strengthened cardiomyocyte cohesion, EGFR does not seem to be required, as was revealed by *Egfr* siRNA knockdown. However, *Egfr* knockdown efficiency, though statistically significant, was weak (31%). Thus, it is possible that the remaining EGFR protein levels were sufficient to mediate the TAPI-1 effect. Nevertheless, EGFR signaling was not affected by TAPI-1 treatment, suggesting that EGFR might not be involved in ADAM17 inhibition-mediated positive adhesiotropy. A recent study in keratinocytes also revealed that ADAM17 inhibition did not alter EGFR signaling (Kugelmann et al., 2022). In mice lacking DSG2, ADAM17 was increased in 2 week old hearts (Ng et al., 2021). Furthermore, increased levels of EGF, indicating an increased EGFR activity, also increased the protein levels of ADAM17 (Santiago-Josefat et al., 2007). However, in our *Jup* ^−/−^ mice, which express low levels of DSG2 and high levels of EGFR (Shoykhet et al., 2022), ADAM17 protein levels were not significantly increased. It has been suggested that the proteolytic activity of ADAM17 is not regulated by protein levels, but by posttranslational modifications (Dusterhoft et al., 2019). We found that in *Jup* ^−/−^ mice, phosphorylation of p38MAPK was increased. Since p38MAPK can activate ADAM17 (Xu and Derynck, 2010; Xu et al., 2012), this might be of interest in understanding the pathogenesis of AC. However, we did not find increased levels of active ADAM17 in *Jup* ^−/−^ mice, nor did we observe increased threonine phosphorylation in lysates pulled down for ADAM17. Indeed, in a previous study, mutating ADAM17 at T735A or deleting its cytoplasmic domain did not affect the activation of ADAM17 by p38MAPK or IL-1β (Hall and Blobel, 2012). Apart from that, in another desmosomal disease, pemphigus vulgaris (PV), an autoimmune condition caused by autoantibodies against DSG1 and/or DSG3, which leads to blister formation in skin and mucosa, p38MAPK is activated, which can be recapitulated *in vitro*. Inhibition of p38MAPK reduced blister formation *in vivo* in mice and in *ex vivo* human skin samples (Berkowitz et al., 2006; Egu et al., 2017). The pathomechanisms of PV and AC are at least in part similar. Indeed, we previously showed that inhibition of p38MAPK in HL-1 cardiomyocytes as well as in *Jup* ^+/+^ and *Jup* ^−/−^ mice enhanced cardiomyocyte cohesion (Shoykhet et al., 2020). Based on previous research and the above findings, it might be that the upregulation of p38MAPK observed in *Jup* ^−/−^ mice activate ADAM17, independent of ADAM17 phosphorylation at T735, resulting in DSG2 cleavage and, thereby a loss of cardiomyocyte cohesion. However, further studies of ADAM17 protease activity in *Jup* ^+/+^ and *Jup* ^−/−^ mice are warranted to address this hypothesis.

Our data suggest that short-term rather than long-term ADAM17 inhibition has a positive adhesiotropic effect on cardiomyocytes, since *Adam17* knockdown or ADAM17 inhibition for 24 h did not result in increased cardiomyocyte cohesion. We hypothesize that other sheddases might be upregulated in response to a longer decreased ADAM17 activity. In fact, it was previously shown that ADAM15 and ADAM9 cleave DSG2 (Klessner et al., 2009).

While ADAM17-deficient mice are not viable, a heart-specific knockdown of *Adam17* led to increased fibrosis and hypertrophy after MI in mice (Fan et al., 2015; Fan et al., 2016). On the other hand, ADAM17 is mediating angiotensin II-induced hypertrophy, which was partly prevented by *Adam17* knockdown or inhibition (Wang et al., 2009; Takayanagi et al., 2016). Inhibition of ADAM17 has been proposed as possible cancer treatment (Saad et al., 2019). In mice with diabetic cardiomyopathy, inhibition of ADAM17 ameliorated fibrosis and apoptosis, whereas ADAM17 deficiency increased cell viability (Xue et al., 2022). Here, we show that acute inhibition of ADAM17 *via* restoring the loss of cardiomyocyte cohesion may be beneficial in AC treatment. The three aspects, that a) inflammation is involved in the pathogenesis of AC (Chelko et al., 2019; Lin et al., 2021), b) ADAM17 causes inflammation (Dusterhoft et al., 2019) and c) our new data, indicate that ADAM17 inhibition could be of therapeutic potential in treating AC. While this approach is unlikely to be suited as a long-term treatment option due to the detrimental effects of prolonged ADAM inhibition (Calligaris et al., 2021), acute inhibition of ADAM17 might be suitable as a treatment to increase cardiomyocyte cohesion in AC.

## Data Availability

The original contributions presented in the study are included in the article/Supplementary Material, further inquiries can be directed to the corresponding author.
