# Supplementary material for: Cardiomyocyte cohesion is increased after ADAM17 inhibition
Source: Front Cell Dev Biol. 2023 Jan 17;11:1021595. doi: 10.3389/fcell.2023.1021595 (PMC9887658; doi:10.3389/fcell.2023.1021595)
Supplement: Supplementary file 1 [file DataSheet1.docx]

Supplementary Material

for

Cardiomyocyte cohesion is increased after ADAM17 inhibition

**Maria Shoykhet^1^, Jens Waschke^1^ and Sunil Yeruva^1*^**

^1^ Chair of Vegetative Anatomy, Institute of Anatomy, Faculty of Medicine, Ludwig-Maximilian-University (LMU) Munich, Munich, Germany


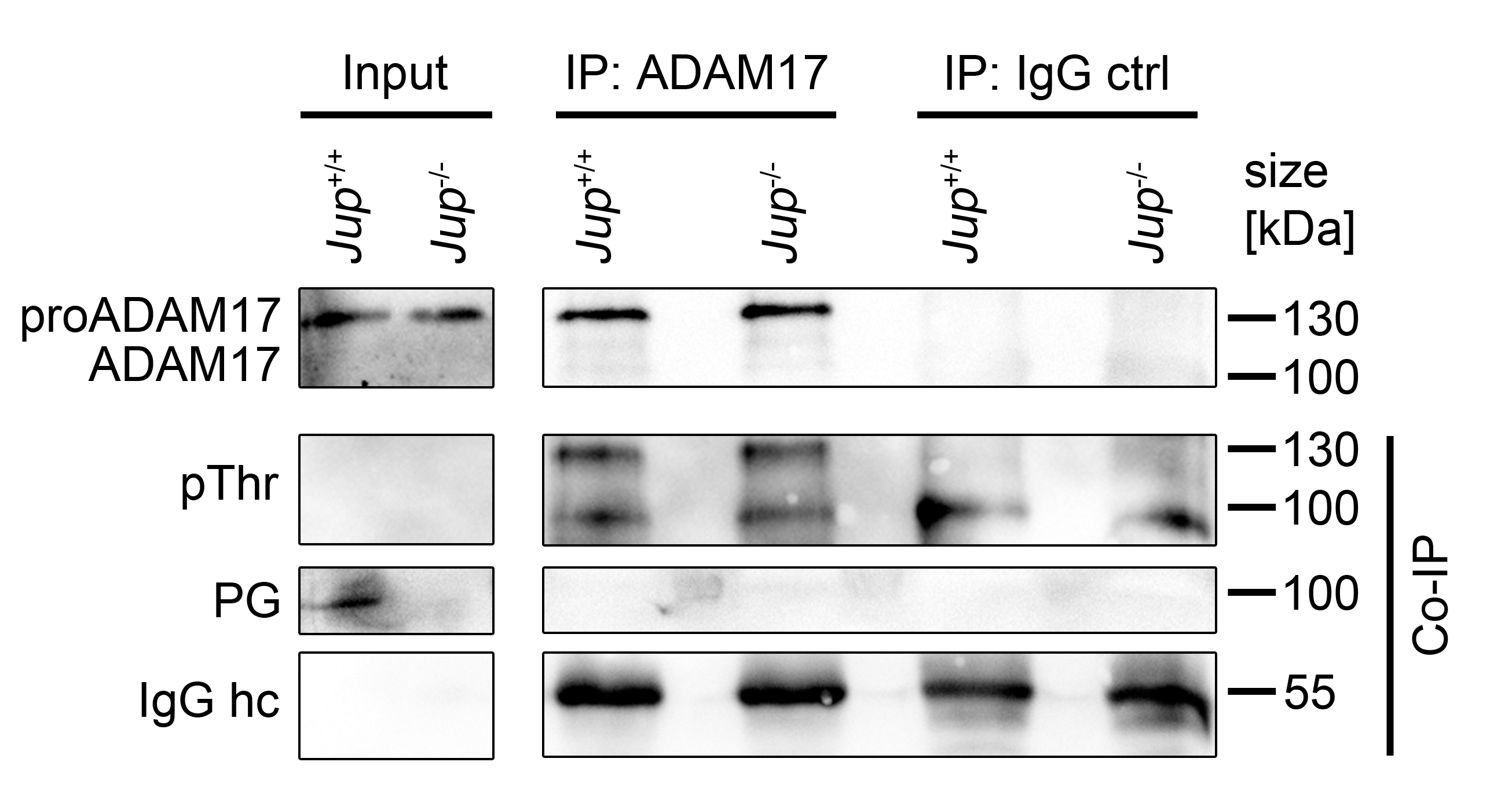


**Supplemental Figure 1.** No changes in ADAM17 phosphorylation between *Jup*^+/+^ and *Jup*^-/-^ mice.

Representative Western blots for immunoprecipitation of ADAM17 with co-immunoprecipitation of phospho-Threonine. IgG heavy chain (IgG hc) served as loading control for immunoprecipitated samples, N = 3.


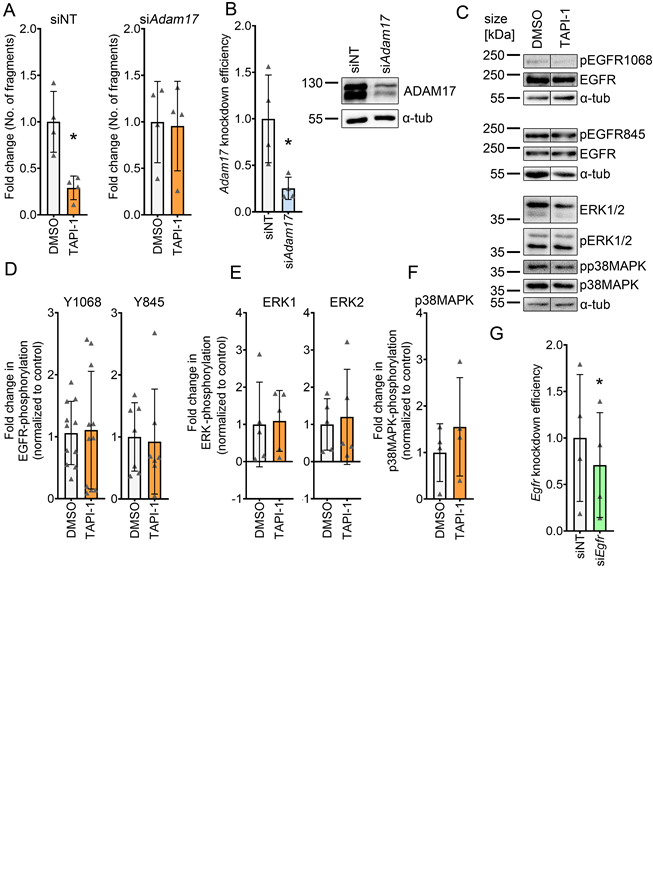


**Supplemental Figure 2.** Effect of TAPI-1 on EGFR signaling

**A**: Dispase-based dissociation assay in HL-1 cardiomyocytes after *Adam17* knockdown and TAPI-1 treatment * p ≤ 0.05, unpaired Student’s *t*-test, N=4. **B**: Representative Western blot confirming *Adam17* knockdown in HL-1 cardiomyocytes with quantification * p ≤ 0.05, paired Student’s *t*-test N = 4. **C**: Representative Western blot of EGFR signaling upon TAPI-1 treatment. Quantification of phosphorylation levels of **D**: EGFR (Y845 and Y1068), **E**: ERK1/2 and **F**: p38MAPK. * p ≤ 0.05, unpaired Student’s *t*-test, N = 4-10. **G**: Quantification of *Egfr* knockdown efficiency, paired Student’s *t*-test N = 4.


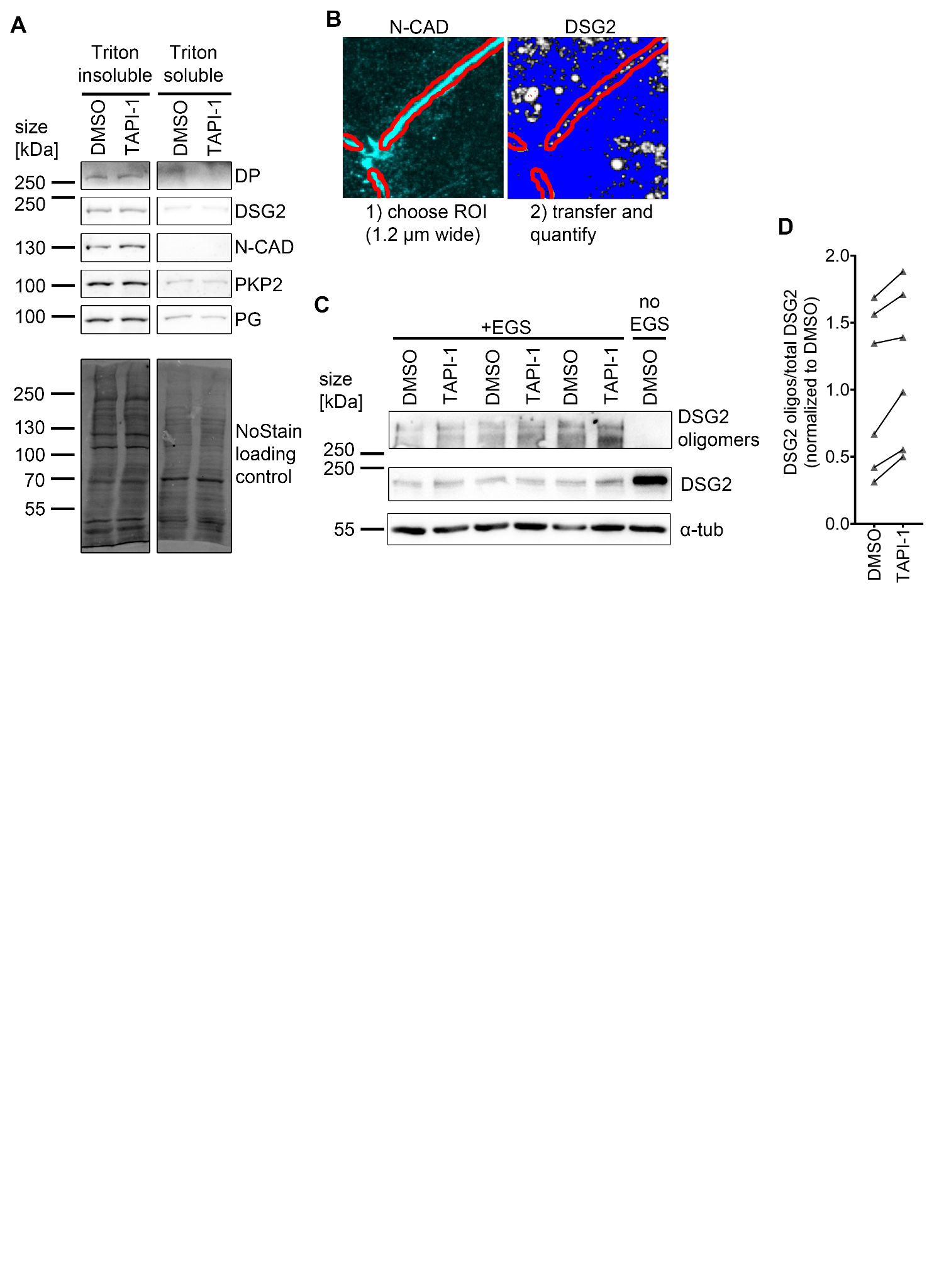


**Supplemental Figure 3.** Effect of ADAM17 inhibition on DSG2

**A**: Representative Triton assay blots for DP, DSG2, N-CAD, PKP2 and PG. NoStain™ dye served as a loading control, N = 3 biological replicates **B**: Exemplary quantification for colocalization in immunostainings. Choice of region of interest in N-CAD image, transfer to DSG2 image and quantification of stained pixels in DSG2 image. A ratio DSG2 to N-CAD at the membrane was calculated and taken as percentage of colocalization of DSG2 in N-CAD regions. **C**: Representative Western blot after EGS crosslinking with and without ADAM17 inhibition for 90 min. No EGS served as control for DSG2 oligomers upon EGS-crosslinking. N = 6. **D**: Quantification of DSG2 oligomerization after EGS crosslinking upon inhibition of ADAM17. N = 6, unpaired Student’s *t*-test.
